# The Role of Dynamic Social Norms in Promoting the Internalization of Sportspersonship Behaviors and Values and Psychological Well-Being in Ice Hockey

**DOI:** 10.3389/fpsyg.2021.744797

**Published:** 2021-11-03

**Authors:** Catherine E. Amiot, Frederik Skerlj

**Affiliations:** ^1^Department of Psychology, Université du Québec à Montréal, Montréal, QC, Canada; ^2^Hockey Lac-St-Louis, Montréal, QC, Canada

**Keywords:** social norms, ice hockey, internalization, psychological well-being, social change

## Abstract

Conducted among parents of young ice hockey players, this field experiment tested if making salient increasingly popular (i.e., dynamic) social norms that promote sportspersonship, learning, and having fun in sports, increases parents’ own self-determined endorsement of these behaviors and values, improves their psychological well-being, and impacts on their children’s on-ice behaviors. Hockey parents (*N* = 98) were randomly assigned to the experimental condition (i.e., presenting dynamic norms that increasingly favor sportspersonship, learning, and fun) vs. control condition (i.e., presenting neutral information). Parents’ motivations for encouraging their child to learn and to have fun in hockey were then assessed. Score sheets for the games that followed the study provided access to their children’s on-ice behaviors (i.e., penalties), as indicators of sportspersonship. Parents in the experimental condition reported higher self-determination for encouraging their child to learn and have fun in hockey compared to parents in the control condition. Furthermore, children of parents in the experimental condition had more assists. A mediation model revealed that the dynamic norms manipulation increased parents’ self-determined motivation for encouraging their child to learn and to have fun in hockey, which in turn, predicted higher psychological well-being (i.e., lower anxiety, more vitality). Together, these results provide support for the contention that highlighting increasingly popular social norms that promote sportspersonship, learning, and fun in sports, represents a promising strategy for creating positive social change in this life context.

## Introduction

The practice of sport can be directly beneficial to well-being and health, and to building positive social relations, including among young people (Bailey, 2006). However, the excessive sense of competitiveness and the pressure that some parents convey to their children are increasingly recognized as important problems in amateur sports (DeFrancesco and Johnson, 1997; Crone, 1999; Fraser-Thomas and Côté, 2009), including in ice hockey (Robidoux and Bocksnick, 2010; Feschuk, 2011; Ackery et al., 2012; Gillis, 2014). This competitiveness undermines team spirit, it harms the general climate within sports teams, and it can generate high levels of stress among parents and their children as well as among coaches, referees, and league administrators (Goldstein and Iso-Ahola, 2006; Bean et al., 2016; see also Lemez et al., 2013). By encouraging ‘winning at all costs,’ this competitiveness can even fuel violent and harmful behaviors in ice hockey (Piedboeuf, 2014). Currently, ice hockey as a sport needs to deal with issues involving its social image, as it has been labeled as a potentially aggressive and dangerous sport (Gatehouse, 2013). This negative image also affects the recruitment of new players and their retention in the sport over the longer-term (MacPhail and Kirk, 2006; Brady, 2011). The media has been argued to contribute to this negative image, by reporting primarily the negative, rather than the positive, social influences and norms that exist in ice hockey (e.g., Pratt, 2020).

To tackle this issue, the sport context needs to find concrete ways to go against the social forces that encourage excessive and unhealthy competitiveness, and to instead promote sportspersonship, wellness, and the healthy development of young players. To this aim, the current study integrates novel approaches on social norms, with the applied considerations, needs, and guidelines stemming directly from sports organizations (e.g., Audet and Dubé, 2017; Hockey Québec, 2017). More specifically, this field experiment tests if making salient increasingly popular social norms that promote sportspersonship (rather than excessive competitiveness) can contribute to this change. The parents of young ice hockey players are targeted in this study given that they play a direct and significant role in their children’s satisfaction and well-being in the context of sports (Scanlan and Lewthwaite, 1986; Petlitchkoff, 1993; Côté, 1999; Côté et al., 2009), as well as on their pro- and anti-sportspersonship behaviors (LaVoi and Babkes Stellino, 2008). We test if the presentation, to ice hockey parents, of social norms that are gaining in popularity – i.e., ‘dynamic’ norms –, and which specifically encourage sportspersonship, learning, and having fun in the context of ice hockey, will increase these parents’ own self-determined (i.e., autonomous) motivation to adhere to these behaviors and values (Ryan and Connell, 1989). And in addition, if these dynamic norms have a beneficial impact on parents’ psychological well-being and on their children’s actual on-ice behaviors.

### The Role of Social Norms

Several established theories and concepts are relevant to understanding how a climate of unhealthy competitiveness can develop in the context of sports, but also how such a situation can be changed. The experimental manipulation employed in the current study builds on one such concept, namely social norms. Social norms refer to what is encouraged and valued in a group (injunctive norm), and what most members of that group do concretely in terms of their behaviors (descriptive norm; Cialdini et al., 1990; Cialdini and Goldstein, 2004).

#### Social Norms in Sports

Social norms play a potent role in the realm of sports. Some norms can promote excessive competitiveness and discourage sportspersonship. For example, sports contexts that normalize and legitimize harmful actions have been associated with increased antisocial behaviors among athletes (Kavussanu et al., 2013). At the intragroup level, the more athletes perceived that their own sports team endorses antisocial behaviors and identified strongly with this team, the more likely they were to engage in these antisocial behaviors themselves, even toward their own teammates (Benson et al., 2017; see also Guivernau and Duda, 2002; Monacis et al., 2014). As well, when football players perceived that their coaches and teammates excuse and condone on-field antisocial behaviors, the more likely they were to personally display antisocial behaviors on the football field (Steinfeldt et al., 2012). In the realm of ice hockey, hockey fans who perceived that derogating the fans of rival (i.e., outgroup) teams is normative and who also identified strongly with other fans of their team were those most likely to engage in derogatory behaviors out of self-determination (Amiot et al., 2014). In contrast, other sports contexts promote sportspersonship and wellness (e.g., Hughes et al., 2016). For example, the more young hockey players perceived that their coaches and parents supported their efforts and mastery of a task (i.e., promoted a task-oriented motivational normative climate; Nicholls, 1984), the more likely these players reported engaging in prosocial behaviors in the context of hockey (Davies et al., 2016; see also Wagnsson et al., 2016).

Because social norms are known to be relative and context-dependent (Turner, 1991; see also Bredemeier and Shields, 1986; Kavussanu et al., 2013), herein we capitalize on this context-sensitive and flexible nature of social norms to investigate how they can be shaped to promote sportspersonship, learning, and fun in sports. In the current work, we rely on an inclusive conceptualization of sportspersonship, which implies: full commitment toward sport participation; respect for traditions and social conventions; respect for the rules and officials; true respect and concern for one’s opponent; and the relative absence of a negative approach to sport participation (Vallerand et al., 1996; Lee et al., 2008).

#### Dynamic Norms

Prior studies and interventions on social norms have typically manipulated descriptive norms (i.e., involving perceptions of which behaviors are typically performed) and injunctive norms (i.e., involving perceptions of which behaviors are typically encouraged and valued) to produce changes in attitudes and behaviors (e.g., Cialdini, 2003). Prior work has also relied on the theory of planned behavior, which includes a normative component, to increase sports participation and physical activity (e.g., Hagger and Chatzisarantis, 2014). In contrast, the current study specifically draws on recent work on changing or *dynamic social norms* to promote sportspersonship, learning, and fun in sports. Dynamic norms are defined as social norms about how other people’s behavior and attitudes are changing over time (Sparkman and Walton, 2017, 2019); such norms hence highlight and encourage novel ways of thinking and behaving (see also Loschelder et al., 2019; Mortensen et al., 2019; Andreoni et al., 2021). Because dynamic norms specifically convey information about collective changes in behavior and attitudes, such norms could directly contribute to promoting social change in a specific life context (Tankard and Paluck, 2016). Importantly, dynamic norms can motivate people to act and to change, despite their prior or current behaviors, and/or the existence of alternative – and sometimes widespread – normative behaviors. For example, experimental research has shown that learning that an increasing proportion of people within one’s ingroup are adopting certain behaviors (e.g., reducing their use of water; reducing meat consumption) influences and motivates individual group members to adopt these growing trends and to intend on changing these behaviors themselves (Sparkman and Walton, 2017).

This dynamic approach to social norms was adopted in the current research not only because of these promising recent findings, but also because leaders and commentators have been increasingly acknowledging the importance of promoting sportspersonship and wellness, and of reducing violence and incivility in sports, including in ice hockey (e.g., Gourd et al., 2016). Building on these theoretical foundations and real-life trends, the proposed research aims to test if making salient real and increasingly popular social norms that specifically promote sportspersonship, learning, and having fun in sports can increase parents’ own autonomous acceptance and internalization of these behaviors and values, and affect their children’s on-ice behaviors.

#### Possible Impact of Dynamic Norms on Parents’ Self-Determination and Psychological Well-Being

While social norms can be seen as a source of (external) pressure and as being controlling, they also have the potential to rationalize and legitimize certain behaviors and values, and to facilitate their (internal) acceptance. Indeed, because they imply that a behavior or attitude is normal or appropriate, norms can convey the message that there are good and shared reasons for engaging in this behavior or for adhering to this viewpoint. In this sense, social norms can facilitate people’s personal endorsement of a behavior and its internalization (Amiot et al., 2012, 2020). Drawing on self-determination theory (SDT; Ryan and Deci, 2017), when this occurs, the normative behavior is likely to be endorsed autonomously and out of choice. Supporting this theoretical contention, the more group members reported personally agreeing with an ingroup norm that encouraged them to engage in a specific behavior, the higher their self-determined motivations for engaging in this normative behavior themselves (i.e., more intrinsic motivation, higher integrated and identified regulations; Amiot et al., 2013). On these bases, we will specifically test if making salient dynamic norms that encourage sportspersonship, learning, and having fun in hockey, will positively impact on parents’ own self-determined motivation for promoting these behaviors and values among their children. At the applied level, uncovering such effects is important to demonstrate that the social norms manipulated can become personally accepted and internalized, a situation which is also likely to yield more sustainable changes over time (Ryan et al., 2008).

Intriguingly, promoting social norms that encourage sportspersonship, learning, and fun in sports (rather than excessive competitiveness) could potentially be beneficial even to the psychological well-being of the players’ parents, who can be quite invested in their children’s sports participation and may experience pressure and anxiety as a result (Fredricks and Eccles, 2005; Horn and Horn, 2007). Indeed, in the motivational literature, prosocial behaviors, such as those that align with sportspersonship values (e.g., respecting and helping others), are theorized to emerge from fulfilled (rather than thwarted) psychological needs and to be associated with higher well-being (Ryan and Deci, 2017; see also Goldstein and Iso-Ahola, 2008). Furthermore, fostering intrinsic motivation – which, by definition, involves experiencing positive emotions (such as having fun) while engaging in a behavior – has been consistently associated with higher psychological well-being and adjustment (e.g., Ryan and Deci, 2000). Similarly, promoting and endorsing mastery goals – which involve a focus on learning and mastering a task according to self-set standards (Nicholls, 1984; Elliott and Dweck, 1988) – has been linked to higher psychological well-being in the realm of sports (e.g., Adie et al., 2008; Harwood et al., 2015). On these bases, we expect that promoting dynamic norms that encourage sportspersonship, learning, and having fun in sports, will create a general mindset that should also have beneficial repercussions on the individual-level well-being of hockey parents.

**FIGURE 1:**
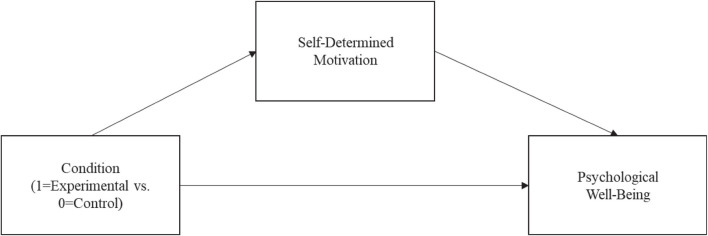
Proposed mediation model testing the role of a self-determined motivation to encourage learning and having fun in hockey in mediating the association between the experimental condition and psychological well-being.

To fully investigate the interplay between dynamic norms, as well as parents’ self-determination and well-being, a mediation model will be tested, as per Figure 1. This model will test if making salient dynamic norms that promote sportspersonship, learning, and fun in sports (vs. making salient neutral information) increases parents’ own self-determined motivation to encourage their children to learn and have fun in ice hockey, and in turn, if this self-determined motivation predicts higher psychological well-being. In other words, if, among hockey parents, a self-determined endorsement of the dynamic norms promoting sportspersonship, learning, and having fun in sports, represents a mechanism through which the dynamic norms manipulation exerts its beneficial impact on well-being. Theoretically and empirically, testing this mediation sequence builds on prior work showing that (external) norms can become (internally) accepted and endorsed autonomously (Amiot et al., 2020); self-determination, in turn, has been consistently associated with higher psychological well-being (e.g., Ryan and Deci, 2017).

#### Possible Impact of Dynamic Norms on Children’s On-Ice Behaviors

Parents can serve as important models for promoting prosociality and sportspersonship among their children, including in ice hockey (Fredricks and Eccles, 2004; LaVoi and Babkes Stellino, 2008). Yet, while sports organizations are required to provide structured and standardized training programs to their coaches (e.g., Coaching Association of Canada, 2021), training programs and interventions that specifically target sports parents are more rare (cf. Respect Group Inc, 2021). Prior intervention studies that have targeted parents have been found to have an impact on their children’s behaviors. For example, in a study conducted among parents of high school students (Harackiewicz et al., 2012), parents in the experimental condition were exposed (via brochures and a website) to information conveying the importance of mathematics and science courses. Students whose parents were in this experimental group then took more sciences and mathematics classes in the last 2 years in high school, compared with the control group. On these bases, the current study will test if making salient, to ice hockey parents, dynamic norms that favor sportspersonship, will impact on the penalties manifested by their children, as an indicator of anti-sportspersonship (Widmeyer and McGuire, 1997; see also Vallerand et al., 1996). Methodologically, including these objective behavioral measures allows to capture real and consequential behaviors and to go beyond self-reported data (Baumeister et al., 2007).

## The Present Study

Correlational studies conducted in the context of sports have consistently shown that social norms predict the endorsement and the manifestation of both prosocial and antisocial behaviors (e.g., Spink et al., 2013; Amiot et al., 2014; Davies et al., 2016; Benson et al., 2017; Bruner et al., 2017). However, research has yet to make salient dynamic social norms that increasingly encourage sportspersonship, learning, and fun in the context of sports, and systematically test these norms’ impact on self-determination, psychological well-being, and on-ice behaviors. To this aim, the current study employs a field-based experimental design to randomly assign parents of young ice hockey players either to: an experimental condition (i.e., that presented real and increasingly popular norms promoting learning, fun, and sportspersonship behaviors) vs. a control condition (i.e., that made salient neutral aspects of hockey: schedule, equipment).

The study was designed in collaboration with the partner organization: Hockey Lac St-Louis, a regional organization located in Québec, Canada, with the goal of finding concrete ways to improve sportspersonship and wellness, and to decrease the excessive competitiveness of ice hockey parents and players. More broadly, this partnership research aligns with Hockey Québec (2017) strategic plan, which seeks to promote the values of learning and having fun among all of hockey players in the Province of Québec. In addition, this strategic plan focuses on promoting a healthy lifestyle through the practice of hockey, on bringing more structure to the involvement of parents in the sport, and on building a sense of belongingness in hockey; these elements are also referred to and embedded in the dynamic norms experimental manipulation employed in the current study. Finally, this strategic plan explicitly recognizes the important changes taking plan in the hockey environment currently, a feature which also ties directly with the dynamic social norms approach and the experimental manipulation employed herein.

In the current study, we specifically test the impact of this manipulation on: (1) parents’ own *self-determined motivation* to encourage their children to learn and have fun in ice hockey; (2) parents’ *psychological well-being*; and (3) their children’s *on-ice behaviors* during the games that followed the study, as concrete and consequential behaviors. In terms of well-being, the current study focuses on two indicators of well-being that are likely to be amenable to a punctual experimental manipulation and which are also relevant in the context of sports, namely: state anxiety and psychological vitality (Woodman and Hardy, 2001; Bartholomew et al., 2011; Amiot et al., 2017). In terms of on-ice behaviors, our main focus is on penalties, as a direct and clear indicator of (anti-) sportspersonship. Access to the score sheets also allows to explore effects on the players’ performance data, namely their goals and assists; the later which represents an indicator of players’ capacity to coordinate with other team members, play as part of a team, and contribute to this team (e.g., Hockey Québec, 2019), and is hence relevant in the general context of sportspersonship.

We expect that:

H1: Parents in the experimental condition will report higher self-determined motivation to encourage their children to learn and have fun in ice hockey compared to parents in the control condition.

H2: Parents in the experimental condition will report lower state anxiety (H2a) and higher vitality (H2b) compared to parents in the control condition.

H3: Children whose parents are in the experimental condition will have less penalties.

H4: A self-determined motivation to encourage one’s child to learn and have fun in ice hockey should mediate the association between the dynamic norms manipulation and the well-being indicators (Figure 1).

## Materials and Methods

### Participants

Emails were sent by the coaches and team managers of the ice hockey teams across the Hockey Lac St-Louis territory who had agreed to take part in the study, to approximately 300 parents of minor league ice hockey players, to invite them to take part in the study. These emails were sent on behalf of the research team. Parents were instructed that only one of the two parents (i.e., the contact parent who had received the email invitation from their child’s team) could take part in the study. The study was presented as investigating the behaviors manifested in ice hockey by young players and their parents, as well as hockey parents’ psychological well-being, also to minimize compliance with the experimental tasks (i.e., minimize demand characteristics). Parents were informed that the study involved completing an online questionnaire along with the tasks and the measures that this questionnaire included, and that the data gathered in this questionnaire would be matched with the score sheets of their child. Participants provided their consent prior to taking part in this study. This study was approved by the researchers’ institutional ethics review board for research with human participants, and was conducted in line with the Canadian Tri-Council Agency Policy for the Ethical Conduct of Research Involving Human Participants. As a compensation for their participation, parents were offered a $10CAN gift certificate. At the end of the research, participants were debriefed about the goals of the study.

Overall, 100 parents took part in the study. Prior to the main analyses, examination of the variables assessed in the questionnaire revealed that none of the participants had univariate outlying scores on more than one variable; however, we excluded two multivariate outliers [Mahalanobis distance: χ^2^(10) = 29.59, *p* = 0.001]. The final sample was comprised of 98 participants. A power analysis conducted in G^∗^Power (Faul et al., 2009), with (1 – β) > 0.80 and α = 0.05 (two-tailed), revealed that this sample size is sufficient to detect a medium effect size (η^2^_*p*_ = 0.06), which had been observed in prior experimental studies on motivation in sports and on changing norms (e.g., Moreno et al., 2010; Mortensen et al., 2019).

#### Parents

Parents comprising the final sample were on average 44.98 years old (*SD* = 5.61; range: 33–60) and 58.2% were mothers and 41.8% were fathers. While 62 participants completed the questionnaire in French, 36 completed it in English; 67.3% of the parents indicated French as their first language, 29.6% had English as their first language, and three participants indicated having another first language (i.e., Filipino, Portuguese). The sample was diverse in terms of highest level of education attained: 13.3% of parents had a high school diploma, 17.3% had attended college, 12.2% had a professional studies diploma, 41.8% had a Bachelor’s degree, 10.2% had a Master’s degree, and one participant had a doctorate (4 participants indicated other degrees: e.g., university-level certificate). It is also worth noting that 25.5% of the parents had played ice hockey in a league themselves (74.5% had not).

#### Children

For data matching purposes (i.e., to match the parent’s online questionnaire with their child’s score sheets), parents were asked to report the name of their child playing in the ice hockey team that was taking part in the current research along with his/her jersey number. Parents were also asked to provide demographic information about this child. These young players were on average 12.38 years old (*SD* = 2.32; range: 8–17.5) and the wide majority of them (93.9%) were boys. The children started playing ice hockey at the age of 5.64 years old on average (*SD* = 1.86; range: 3–11). In terms of age categories, 22.4% of the young players were playing in the Atom category, 23.5% were in Pee-Wee, 40.8% were in Bantam, and 13.3% were in the Midget category. Children played either as forwards (49%), defense (37.8%), or goalies (13.3%). In terms of the competitive level at which the children played, 20.4% played in a Tier 1-level team (AAA; most competitive), 40.9% played in a Tier 2 team (AA, or BB), and 38.7% played in a Tier 3 team (A, B, or C; least competitive).

### Design and Procedure

This study employed a between-participants design comprised of two conditions: the experimental and the control condition. The study involved several consecutive steps. In the online questionnaire, parents first provided demographic information and information about their child (e.g., competitive level/age category/position played, jersey number). Then, parents were randomly assigned either to the experimental (*n* = 52) or to the control (*n* = 46) condition. The Qualtrics platform used for the current study allowed to embed the manipulations within the online questionnaire and to randomly assign participants to these two conditions. Following this experimental manipulation, the parents then completed a measure assessing their motivations to encourage their child to learn and have fun in ice hockey, followed by measures that assessed their psychological well-being. At the end of the questionnaire, an open-ended question asked parents to indicate what were the goals of the study in their view; no participants had guessed the specific goal of this study (i.e., which was to test if social norms that promote sportspersonship, learning, and fun in ice hockey impact on parents’ motivation and psychological well-being, and on their children’s on-ice behaviors), and no participant explicitly mentioned being aware of the existence of the two conditions that were present in this study: i.e., experimental and control. Finally, the score sheets for the hockey games, played by the child, that followed the completion of their parent’s questionnaire were obtained from the hockey teams and coded by the research team. Together, these procedures allowed us to combine and to match the data obtained from parents in the online questionnaire, with the on-ice behavioral data of their children. The online questionnaire was completed at the start of the ice hockey season (i.e., from mid-October to early November 2018), as a relevant moment to intervene in the sports season (Audet and Dubé, 2017).^1^

#### Experimental Condition

Based on established procedures (Marigold et al., 2007; Cohen et al., 2009; Paluck, 2011; Walton and Cohen, 2011; Finkel et al., 2013), parents in the experimental condition were asked to complete seven writing and reading tasks. These tasks specifically aimed to make salient the increasingly popular (i.e., dynamic) social norms that favor sportspersonship and healthy development in sports, and to promote participants’ own appropriation and internalization of these norms. Because norms that are endorsed by a diversity of social groups tend to have a stronger impact on people’s attitudes and behaviors (e.g., Verkooijen et al., 2007), and to maximize the impact of the manipulation, the norms presented across the tasks of the experimental condition originated from different sources, namely: sports analysts and journalists, elite ice hockey players as role models, and other hockey parents that participants know.

For the *first task* (adapted from Sparkman and Walton, 2017), parents in the experimental condition read a text presenting real facts about how norms in ice hockey are changing, and how different social actors (e.g., sports analysts and journalists, elite hockey players) are increasingly promoting the benefits of having fun, learning, and valuing sportspersonship, rather than excessive and unhealthy competitiveness. This text was as follows: “The world of hockey is changing at the moment. In recent years, people have become more and more aware of the importance of a positive climate in sports and are valuing learning and cooperation instead of ‘winning at all costs.’ Several recent examples illustrate this change in mentality, in hockey well as in other sports: the documentary *Parents Inc*, which illustrates how competitiveness can hinder the development of young hockey players and even lead them to drop out of their sport. The movie *Concussion* shows how aggressive behaviors in sports can be detrimental to human health and can be profoundly harmful to football players. Dany Dubé and Isabelle Audet’s recent book encourages athletic achievement through pleasure, learning, and the development of good relationships within the team, rather than through unhealthy competitiveness. Hockey Québec has now banned cross-checking in certain categories. In the Québec Major Junior Hockey League, the number of major penalties has dropped by nearly half from 2013 to 2018. Several high-level hockey players believe that in order to perform well and to reach the higher levels in their sport, it is important that players show strong team spirit rather than unsportsmanlike aggressivity. These players also claim that it is important to concentrate on one’s own goals and to set controllable and reachable objectives. Therefore, there are profound changes in mentality emerging in hockey and these changes are growing in popularity.”

In the *second task* (adapted from Walton and Cohen, 2011), and to reinforce these norms, parents in the experimental condition were asked to write three sentences that illustrate why, in their opinion, people increasingly believe in the importance of promoting team spirit and respect in the context of ice hockey, and how their own experience aligns with this trend. As a *third task* (adapted from Marigold et al., 2007), parents were asked to pick one out of four testimonials representing examples of other parents who encourage sportspersonship, learning, and fun in the context of ice hockey, and to write about how this testimonial aligns with their own reality and family life. These testimonials were as follows:

(1) David is the father of a boy who plays as a winger in bantam CC this year. David believes that participating in sports allows young people to learn things that will be useful to them in their life in general. He does his best to keep this in mind, even when it is tough and things are not going well during a game.
(2) Jean-Nicolas is the father of a girl who is a goalie at the novice A level. Jean-Nicolas encourages his daughter to set goals that will allow her to have fun in hockey and to improve gradually, without putting too much pressure on her.
(3) Corinne is the mother of a boy who plays as a defense in midget AA this year. Corinne values team spirit a lot. When watching her son cheering and supporting his teammates during tight and tense games, she always takes the opportunity to let her son know how much this kind of behavior will take him far in life and will also help him face challenges.
(4) Hubert is the father of a boy playing at the pee-wee B level. His son hates to lose a game. However, when this happens, Hubert tries to put things into perspective for his son. He does so by helping his son focus on what he did right despite the failure and set controllable and realistic goals for the next game.

As a *fourth task* (adapted from Cohen et al., 2009), parents were asked to indicate whether (Yes vs. No): ‘Having fun should be more important than winning at all costs’; ‘Learning should be an important value in hockey’; and ‘Good relations with teammates should be more important than aggressivity.’ Also to reinforce the salience of the pro-sportspersonship norms in ice hockey, in the *fifth task* (adapted from Marigold et al., 2007; Cohen et al., 2009; Finkel et al., 2013), they were asked to identify concrete examples of behaviors that parents of other ice hockey players do to: encourage their child; cooperate with the coach; highlight their child’s efforts after a demanding game or practice; change the negative climate during or after a game that does not go well. In the *sixth task*, they were asked to provide examples for these same behaviors, but that they had manifested themselves. In the *seventh task* (adapted from Walton and Cohen, 2011; see also Paluck, 2011), parents were asked to provide an advice to another parent on how to encourage their child to focus on their own (personal, team) goals rather than on ‘winning at all costs.’

#### Control Condition

Participants in the control condition performed seven tasks of the same formats and lengths. However, these tasks focused on neutral aspects of ice hockey, which were unrelated to sportspersonship, learning, and fun. Specifically, these tasks focused on the hockey schedule (i.e., the possible upcoming changes that will be brought to this schedule and the role of parents in managing their child’s hockey schedule), and on their child’s ice hockey equipment (i.e., the role of parents in choosing their child’s equipment and in helping their child care for their equipment).

### Measures

#### Parents’ Self-Reported Measures

After completing the seven tasks, parents in both conditions completed measures assessing their motivation and well-being, including measures of state anxiety and psychological vitality.^2^

The six items assessing *parents’ motivation to encourage their child to learn and have fun* in ice hockey were based on the SDT continuum; these items were adapted from prior established motivation scales (i.e., Ryan and Connell, 1989; Guay et al., 2003) and from a measure assessing self-determined and non-self-determined motivations in ice hockey (Amiot et al., 2014). As such, these items were relevant to the specific context under investigation, while also being theoretically based. Participants were first presented with the following stem: “Now, think about the reasons that can motivate you to encourage your child to learn and to have fun (rather than to be very competitive) in the context of hockey. Using the response scale, indicate to what extent each of the items below refers to the reasons why you encourage your child to focus on learning and having fun in hockey.” A 7-point Likert scale was used (ranging from 1 = *Not at all* to 7 = *Completely*). Three items assessed the *self-determined (i.e., autonomous) motivations* (α = 0.64) of: intrinsic motivation (“Because I derive satisfaction from acting this way”), integrated regulation (“Because doing so corresponds to my personal values”), and identified regulation (“Because focusing on learning and enjoyment helps to achieve important goals in life”). Three items assessed the *non-self-determined (i.e., controlled) motivations* (α = 0.58) of: introjected regulation (“Because I have to act that way to feel that I am a person of worth”), external regulation (“Because doing so allows me to be recognized socially by others”), and amotivation (“I wonder why I even encourage my child to learn and have fun; in fact, I think it is useless”).

To capture parents’ situational well-being, we first measured *state anxiety* (α = 0.86) using the six items from the short-form of the State-Trait Anxiety Inventory (Marteau and Bekker, 1992). Participants were asked to rate the feelings that they were currently experiencing (e.g., “I feel calm”), on a 1 (*does not correspond at all*) to 7 (*corresponds exactly*) scale. *Vitality* (α = 0.91), a well-being indicator that captures its energization component, measures the extent to which a person feels alive and energetic (Ryan and Frederick, 1997). Participants were asked to indicate the extent to which each of the items corresponded to how they were feeling. The six items of this scale (e.g., “I feel alive and vital”) were rated on a 1 (*does not correspond at all*) to 7 (*corresponds exactly*) scale.

#### Children’s On-Ice Behaviors

Score sheets were obtained from the ice hockey teams; the current analyses focused on the two games played just after the parents had completed the online questionnaire (i.e., these games were played within 10 days following the questionnaire), a timeframe considered appropriate given the nature of the online manipulation and also likely to be affected by this manipulation. Trained scorekeepers filled-in the score sheets during the hockey games; they had each received a formal training session (of 8 h) prior to the start of the hockey season (plus a yearly refresher course), informing them about how to complete these score sheets correctly during the games. Each score sheet documented the duration and types of penalties observed for each player per game, as well as the number of goals and assists per player (Hockey Québec, 2018). We obtained the score sheets for 80 players. These score sheets were coded by trained research assistants and three variables were created, representing: (1) the total duration (in minutes) of penalties during these games, (2) the total number of assists, and (3) the total number of goals during these games. Goalies (*n* = 10) were excluded from the analyses that involved the variables pertaining to the number of penalties, goals, and assists (see also Banse et al., 2015). During these games, a total of 24 penalties were issued [i.e., for players who received a penalty(ies), the range was: 2–62 min], 14 players had at least one assist [i.e., for those who had an assist(s), the range was: 1–3 assists], and 11 players scored at least one goal [i.e., for those who scored a goal(s), the range was: 1–3 goals]. The penalties observed pertained to aggressive (e.g., fighting), non-aggressive (e.g., interference), and other anti-sportspersonship behaviors (e.g., yelling at officials; Widmeyer and McGuire, 1997); none of the penalties pertained to the use of non-standard equipment.

## Results

### Descriptive Statistics and Correlations

Table 1 presents the associations among the parents’ self-reported measures (i.e., motivations and well-being) and their children’s on-ice behaviors. As seen in Table 1, a self-determined motivation for encouraging one’s child to learn and to have fun in hockey was associated with lower anxiety but with higher vitality. In terms of the behavioral on-ice variables, parents’ anxiety was marginally associated with their children scoring fewer goals during the games that followed the presentation of the experimental manipulation.

**TABLE 1:** Bivariate correlations among the psychological variables and the on-ice behavioral measures.

	***M***	***SD***	**2**	**3**	**4**	**5**	**6**	**7**
(1) Self-Determined Motivation	5.95	0.97	0.12	–0.29**	0.23**	0.02	0.07	–0.07
(2) Non-Self-Determined Motivation	1.72	1.03	–	0.03	–0.002	–0.09	0.01	0.18
(3) State Anxiety	2.49	1.05		–	–0.50***	0.01	0.12	–0.20^†^
(4) Vitality	5.47	0.97			–	–0.12	–0.05	–0.09
(5) Penalty Duration (in minutes)	1.80	7.45				–	–0.01	–0.01
(6) Assists	0.48	0.79					–	0.08
(7) Goals	0.34	0.74						–

^†^p < 0.10; *p < 0.05; **p < 0.01; ***p < 0.001.

*The correlations involving the variables pertaining to number of penalties, assists, and goals exclude goalies.*

### Analyses of Variance

When testing, using between-participants ANOVAs, whether the manipulation had an effect on parents’ motivation to encourage their children to learn and have fun in hockey and on their well-being, and as can be seen in Table 2, parents in the experimental condition indeed demonstrated higher levels of self-determined motivation than parents in the control condition. This result supports H1. However, we did not observe significant differences on the two well-being indicators, which does not support H2a or H2b, nor on the non-self-determined motivation variable. On the on-ice behavioral variables, we found that children of parents in the experimental condition demonstrated significantly more assists than children of parents in the control condition. There was also a tendency for the children in the experimental condition to have a lower duration of penalties (*p* = 0.269, η*^2^*_*p*_ = 0.02), in line with H3; the lack of a significant effect on this variable, despite the means observed for each of the two conditions (i.e., *M* = 0.86 for the experimental and *M* = 2.85 for the control condition), could be due to the particularly large variability in penalties observed in the control condition (i.e., *SD* = 10.72).

**TABLE 2:** Comparison of the experimental and control conditions on the psychological variables and the on-ice behavioral measures.

	**Experimental condition**		**Control condition**					
	***M***	***SD***	***M***	***SD***	***dfs***	***F***	**η ^2^_*p*_**	***p***
Self-Determined Motivation	6.17	0.83	5.70	1.07	1, 96	5.83	0.06	0.018
Non-Self-Determined Motivation	1.72	0.76	1.72	1.29	1, 96	0.00	0.00	0.993
State Anxiety	2.39	0.98	2.59	1.12	1, 94	0.85	0.01	0.360
Vitality	5.51	1.05	5.44	0.88	1, 95	0.12	0.001	0.728
Number of Assists	0.68	0.91	0.27	0.57	1, 68	4.74	0.07	0.033
Penalty Duration	0.86	1.53	2.85	10.72	1, 68	1.24	0.02	0.269
Number of Goals	0.27	0.65	0.42	0.83	1, 68	0.75	0.01	0.389

*The analyses involving the variables pertaining to number of penalties, assists, and goals exclude goalies.*

### Mediation and Indirect Effects

While the experimental manipulation did not impact on the well-being measures, the ANOVA revealed a significant difference in self-determined motivation between the conditions; self-determination, in turn, was associated with significantly lower anxiety and higher vitality. Based on this pattern of findings, and given that current thinking about mediation analysis does not require the presence of a statistically significant total effect (whereby the independent variable has a significant effect on the dependent variable) for estimating indirect effects (Hayes, 2018), we proceeded to tests of mediation and indirect effects. These tests allow to establish if the manipulation affected parents’ well-being, specifically through an increase in their self-determined motivation. Using the PROCESS macro for SPSS (Hayes, 2018), Model 4 of this macro tests the indirect effect of the predictor on the dependent variable through a mediator based on a bootstrap analysis, which estimates confidence intervals around the indirect effect. For the present analyses, we relied on 95% percentile bootstrap intervals with 5000 bootstrap samples.

**FIGURE 2:**
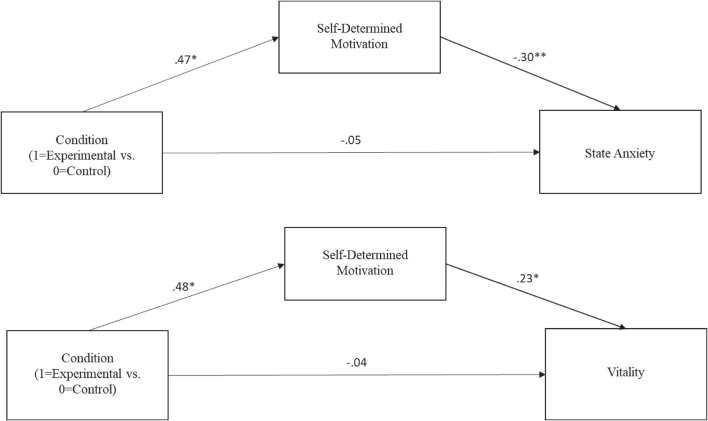
Path coefficients observed in the two mediation models tested (*p < 0.05; **p < 0.01).

Two models were estimated, in line with H4. In the first model, and as can be seen in Figure 2, the condition (experimental vs. control) served as an independent variable, self-determined motivation as the mediator, and state anxiety as the dependent variable. Results showed that being in the experimental condition predicted higher self-determined motivation to encourage one’s child to learn and have fun in hockey (*b* = 0.47, *SE* = 0.20, *p* = 0.0172; 95% CI [0.09; 0.86]). In turn, this self-determined motivation predicted lower anxiety (*b* = –0.30, *SE* = 0.11, *p* = 0.0073; 95% CI [–0.52; –0.08]). There was a significant indirect effect of the condition on anxiety through self-determined motivation (IE = –0.14, *SE* = 0.07, 95% CI [–0.30; –0.02]). The direct effect of the condition on anxiety was not significant, however (*b* = –0.05, *SE* = 0.21, *p* = 0.7980, 95% CI [–0.48; 0.37]).

In the second model, the condition (experimental vs. control) also served as the independent variable and self-determined motivation as the mediator, whereas vitality was the dependent variable (see Figure 2). Again, being in the experimental condition predicted higher self-determined motivation to encourage one’s child to learn and have fun in hockey (*b* = 0.48, *SE* = 0.19, *p* = 0.0154; 95% CI [0.09; 0.86]). In turn, this self-determined motivation predicted higher vitality (*b* = 0.23, *SE* = 0.10, *p* = 0.0286; 95% CI [0.02; 0.43]). There was a significant indirect effect of the condition on vitality through self-determined motivation (IE = 0.11, *SE* = 0.06, 95% CI [0.01; 0.25]). The direct effect of the condition on vitality was not significant (*b* = –0.04, *SE* = 0.20, *p* = 0.8417, 95% CI [–0.44; 0.36]). Thus, the impact of the manipulation on the two well-being outcomes operated indirectly, through parents’ own self-determined motivation to encourage their child to have fun and to learn in hockey. These findings provide some support for H4.

## Discussion

Conducted among parents of young ice hockey players, the objective of this study was to investigate if making salient increasingly popular social norms that promote sportspersonship, learning, and fun in sports (rather than excessive competitiveness) increases parents’ own self-determined (i.e., autonomous) motivation to endorse such behaviors and values. We also tested if these dynamic norms have a beneficial impact on parents’ psychological well-being and on their children’s on-ice behaviors. To this aim, the current study integrated the needs and concerns of sports organizations and commentators (e.g., Audet and Dubé, 2017; Hockey Québec, 2017), with novel theoretical approaches on social norms – namely, research on dynamic or trending norms (e.g., Sparkman and Walton, 2017, 2019; Mortensen et al., 2019). Indeed, a growing literature reveals that making salient social norms that are becoming increasingly popular motivates people to align their own behaviors and attitudes with these emerging trends. Parents of players were targeted by this manipulation given their capacity to influence their children’s behaviors, in sports (e.g., Côté, 1999; LaVoi and Babkes Stellino, 2008) and in other life contexts (e.g., academic: Harackiewicz et al., 2012).

A field-based experimental design was employed. Specifically, participants were randomly assigned either to an experimental condition, which presented actual and increasingly popular norms that promote sportspersonship, learning, and having fun, vs. to a control condition. Doing so allowed to rely on a systematic yet context-relevant method to test our research hypotheses (Branscombe and Wann, 1994; Wann, 2006). Parents’ own autonomous (i.e., self-determined) motivation for endorsing these behaviors and values was then measured, along with their levels of well-being. Their children’s one-ice behaviors in the games that followed the experimental manipulation were obtained through score sheets, which allowed access into real and consequential behaviors (Baumeister et al., 2007).

We expected that parents in the experimental condition would be more personally motivated – i.e., more self-determined – to encourage their children to learn and to have fun in hockey compared to parents in the control condition (H1), and would also report higher psychological well-being following the experimental manipulation, namely lower state anxiety (H2a) and higher vitality (H2b). Children whose parents were in the experimental condition were also expected to have less penalties on-ice, as an indicator of anti-sportspersonship (H3). Finally, and in line with prior research showing that norms can promote a self-determined endorsement of the normative behaviors, and that self-determination in turn, consistently predicts higher psychological well-being (Ryan and Deci, 2017), we expected that parents’ self-determined motivation to encourage their child to learn and have fun in ice hockey should mediate the association between the experimental manipulation and the two well-being outcomes (H4).

Results from the ANOVAs provided support for H1: Parents in the experimental condition reported higher self-determined motivation for encouraging their child to learn and to have fun in hockey compared to parents in the control condition. This finding provides evidence for the contention that making salient relevant and increasingly popular (i.e., dynamic) social norms can promote the internalization – in the form of higher self-determination – of the values and behaviors promoted by these norms (e.g., Amiot et al., 2020). It should be noted that the experimental manipulation had no effect on parents’ non-self-determination; this finding suggests that making salient dynamic norms that increasingly promote learning, sportspersonship, and having fun, does not impact on the more controlled (i.e., non-self-determined) forms of motivations and, in this sense, does not seem to have been interpreted as pressuring by the participants (e.g., Amiot et al., 2013, 2020).

With respect to H2a and H2b, no significant differences emerged on the situational well-being levels of parents in the experimental vs. in the control condition, suggesting that promoting a normative climate that encourages learning, fun, and sportspersonship does not appear to be sufficient to trigger higher well-being among hockey parents. However, tests of mediation and indirect effects revealed that the impact of the experimental manipulation on parents’ psychological well-being operated indirectly, through these parents’ own self-determined motivation for encouraging their child to learn and have fun in hockey. These findings provide some support for H4. Conceptually, they imply that it is imperative that parents personally and autonomously accept (i.e., internalize) external norms in favor of sportspersonship, leaning, and fun, for this internalization to then flow on to predict higher well-being. In other words, in the current study, self-determination was found to be a necessary process that enabled social norms to positively predict individual-level well-being. This finding is important both from a social norms and from an applied perspective; it shows that for social norms to truly be beneficial to well-being, individual group members need to autonomously accept the social behaviors and the values that these norms promote, and to consider that these behaviors and values are meaningful and personally important to them (see also Ryan et al., 2008).

In line with SDT tenets, the current study uncovered significant associations between a self-determined motivation for encouraging one’s child to learn and to have fun in hockey and psychological well-being (i.e., lower anxiety, higher vitality). It is important to note that these associations may also operate reciprocally, and that parents’ motivation in real life could be associated with their motivation to encourage their children positively in sport. Future research should be designed to capture these reciprocal directions and top-down processes, whereby individuals’ general (i.e., global) motivation impacts their motivation in a specific context or situation (e.g., Vallerand, 1997).

In terms of on-ice behaviors, children whose parents were in the experimental condition had an average penalty duration of 0.86 min, whereas children whose parents were in the control condition had an average penalty duration of 2.85 min. Although this difference was not statistically significant, these means point to a potentially beneficial role for the dynamic norms manipulation in decreasing players’ anti-sportspersonship behaviors on the ice, in line with H3. In terms of on-ice performance, while no significant differences were found in terms of goals, children whose parents were in the experimental condition had significantly more assists compared to those whose parents were in the control condition. This last finding suggests that the dynamic norms manipulation, which promoted sportspersonship and which highlighted, among other elements, the importance of team spirit and collaboration among players, positively impacted the players’ assists, as an on-ice behavior that also captures players’ capacity to play as part of a team and contribute to this team. Taken together, these performance findings are important in showing that making salient social norms that increasingly promote sportspersonship, learning and fun, does not impede performance in terms of the number of goals scored (i.e., such norms do not ‘harm’ performance nor ‘demotivate’ the players). Instead, such norms positively contribute to a more collaborative and collective indicator of performance; i.e., more assists. It should be noted that the use of score sheets to capture players’ on-ice behaviors as its limitations and includes measurement error, as with any type of measure. Whereas officials (who assign penalties) occupy a technically impartial role in ice hockey games and experience high pressure to fulfill this role, they are not immune to making mistakes. Similarly, scorekeepers are trained to transcribe the penalties and points issued during a game; yet they can also make errors (e.g., reporting the wrong penalty code on the score sheet).

### Contribution, Limitations, and Future Research

The current study builds on prior work on dynamic norms (e.g., Sparkman and Walton, 2017, 2019) as well as human motivation (e.g., Ryan and Deci, 2017) by specifically showing that hockey parents can autonomously accept increasingly popular social norms that favor sportspersonship, learning, and fun, and that this normative manipulation also has beneficial implications for their children’s actual observable on-ice behaviors (i.e., greater number of assists). The use of score sheets in the current research allows us to go beyond self-reported data. While these findings are encouraging, future research should systematically investigate if these associations can be consolidated over longer periods of time, using multimodal interventions for instance. Indeed, interventional designs are increasingly used in sports psychology and in psychology more generally (e.g., Walton, 2014; Camiré et al., 2020). Yet, caution is needed when designing such future interventions.

First, interventions that seek to activate and to promote certain social norms need to be implemented mindfully and directly grounded into the existing literature, or else they run the risk of backfiring (e.g., Smith and Louis, 2009). For example, if many other hockey parents say that they value sportspersonship in sports (i.e., they endorse an *injunctive norm* that favors sportspersonship), but in actuality, these parents do not behave in line with the principles of sportspersonship (i.e., the *descriptive norm* goes against sportspersonship), this situation is likely to elicit a normative conflict between injunctive and descriptive norms. Such a normative conflict is then likely to be demotivating, and to discourage parents to behave in line with sportspersonship values (see Smith and Louis, 2009). In the current research, we elected to use a norms manipulation grounded in the dynamic norms literature; doing so allowed us to circumvent this potential backfiring effect of norms (see also Sparkman et al., 2021). Indeed, regardless of which specific proportion of parents actually value and align their behaviors with the principles of sportspersonship, the fact that an *increasing* number of people are adhering to this growing trend should be, in itself, motivating.

Second, durable human change may take time to generate and to observe. Whereas the current study employed a relatively short-term manipulation (i.e., 15–20-min long), more intensive interventions could also be designed to produce longer-term changes over time. For example, using a combination of both micro-level cognitive behavioral strategies and macro-level institutional changes, such as by modifying the mandatory rules of the game and punishing anti-sportspersonship more harshly, could prove particularly useful for generating such changes (see Cusimano et al., 2013). In line with these propositions, Hockey Québec has developed different interventions aimed at reducing anti-sportpersonship behaviors through social participation, and which specifically target and involve hockey parents (e.g., Equijustice, 2019). Such interventions also align with the values put forward in the Hockey Québec (2017) strategic plan (e.g., having fun, increasing a sense of belonging within the hockey community).

Future work should also directly identify the developmental factors that operate in the associations observed herein (e.g., Gatto et al., 2010; Rutland et al., 2010). For instance, observational studies could be conducted to capture how exactly parents’ values and observable behaviors change following a normative intervention (e.g., Blair et al., 2019), and come to trickle-down and affect their children’s own behaviors and values in sports. These future studies could also capture the specific and non-defensive strategies through which parents come to convince those around them that highly competitive norms need to change and be replaced with norms that promote sportspersonship instead (Bastian, 2019). Such research would provide further insights into how, concretely, dynamic norms can contribute to generate social change (Täuber and Sassenberg, 2012; Tankard and Paluck, 2016). Using systematic observation, this future work could capture and assess quite subtle behaviors (e.g., non-verbal behaviors, body language), as these behaviors also directly contribute to the transmission of social messages and norms (e.g., Dovidio and Gluszek, 2012; Mehrabian, 2017), as well as include observable indicators of psychological well-being (e.g., anxiety). These observations could be particularly relevant to make in contexts of high stress or adversity (e.g., following a goal scored by the opposing team).

In keeping with the applied focus of the current research, future studies should also be designed to systematically investigate if anti-sportspersonship behaviors are more prevalent in some groups of ice hockey players than others (e.g., older players, players from more competitive levels), and whether the current dynamic norms manipulation works more efficiently in some of these groups compared to others. Finally, sportspersonship could be assessed, in future research, using a multi-method approach; for example, by also including systematic observations of a wider range of both pro- and anti-sportspersonship behaviors (both on ice and in other life contexts such as at home), self-reported measures of sportspersonship, and content analysis of both parents’ and players’ exchanges on social networks. Doing so would provide valuable additional information about the level of sportspersonship behaviors actually manifested, across different methods and life contexts.

In sum, this paper brings together emerging work on social norms with the applied considerations, recommendations, and needs of sports organizations, to investigate an important and timely question: how the sports context can be structured so as to promote sportspersonship, wellness, and the healthy development of young players. This question was investigated in a field experiment conducted among sports parents in an involving and socially relevant context. Future research employing more intensive designs and further testing the mechanisms that are at play will be useful to inform the development of longer-term, multimodal interventions seeking to promote positive social change in the realm of sports.

## Data Availability Statement

The raw data supporting the conclusions of this article will be made available by the authors, without undue reservation.

## Ethics Statement

The study involving human participants was reviewed and approved by Université du Québec a Montréal ethics board for research conducted with human participants. Written informed consent to participate in this study was provided by the participants’ legal guardian/next of kin.

## Author Contributions

CEA and FS contributed to conception and design of the study. FS facilitated the logistics of participants recruitment. CEA organized the database, conducted the statistical analysis, and wrote the first draft of the manuscript. Both authors contributed to manuscript revision, read, and approved the submitted version.

## Conflict of Interest

The authors declare that the research was conducted in the absence of any commercial or financial relationships that could be construed as a potential conflict of interest.

## Publisher’s Note

All claims expressed in this article are solely those of the authors and do not necessarily represent those of their affiliated organizations, or those of the publisher, the editors and the reviewers. Any product that may be evaluated in this article, or claim that may be made by its manufacturer, is not guaranteed or endorsed by the publisher.

## Footnotes

1 These data are part of a larger dataset that included a longitudinal component and two subsequent timepoints. This is the only manuscript that presents data from this dataset; no prior published work has presented nor used the data (in whole or in part) from this research. Because of the high attrition rates observed at these following timepoints (e.g., up to 55% for the online questionnaire), and the associated loss of statistical power, the data from these two timepoints are not presented in the current manuscript. More information about these subsequent timepoints can be accessed via the following link: https://osf.io/2pyk8/?view_only=ed731d197cf243baaf2ce53c86a26a89

2 Additional scales were included in the online questionnaire. Specifically, the *Inclusion of the Team in the Self* [adapted from the Inclusion of the Other in the Self Scale (IOS); Aron et al., 1991] was included as a baseline measure prior to the manipulation, also to ascertain that the two conditions did not differ in terms of their feelings of connection with the ice hockey team of their child. The IOS is a pictorial scale that assesses the degree of closeness between the self and others. It includes seven Venn diagrams with varying degrees of overlap between the circles and which range from 1 (*least overlapping*) to 7(*most overlapping*). In this study, one circle represented the self (“me”), while the other circle represented the child’s team. Parents were instructed to select the diagram that best described the relationship between themselves and their child’s hockey team. An ANOVA confirmed that there was no significant difference between the experimental (*M* = 4.38, *SD* = 1.88) and the control conditions (*M* = 4.70, *SD* = 1.78) on this measure [*F*(1,96) = 0.70, *p* = 0.404, η^2^_*p*_ = 0.01]. This variable also showed only one significant correlation with the dependent variables (i.e., with vitality: *r* = 0.21, *p* = 0.042). As well, following the manipulation, additional, and non-situational, measures of well-being were taken, specifically: Rosenberg’s (1965) self-esteem scale; life satisfaction (Diener et al., 1985); meaning in life and search for meaning (Steger et al., 2006); and positive relationships with others (Ryff, 1989). The experimental manipulation did not significantly impact on these well-being measures (*F*s ranged between 0.62 and 1.78; *p*s between 0.185 and 0.433; and η*^2^*_*p*_s between 0.006 and 0.018).
